# Prevention of atrial fibrillation with SGLT2 inhibitors across the spectrum of cardiovascular disorders: a meta-analysis of randomized controlled trials

**DOI:** 10.1093/ehjcvp/pvaf040

**Published:** 2025-06-04

**Authors:** Damiano Fedele, Marcello Casuso Alvarez, Angelo Maida, Nicolò Vasumini, Sara Amicone, Lisa Canton, Michele Di Leo, Marco Basile, Tommaso Manaresi, Francesco Angeli, Matteo Armillotta, Luca Bergamaschi, Carmine Pizzi

**Affiliations:** Department of Medical and Surgical Sciences (DIMEC), Alma Mater Studiorum, University of Bologna, Via Zamboni, 33, Bologna 40126, Italy; Cardiovascular Division, Morgagni-Pierantoni University Hospital, Via Carlo Forlanini, 34, Forlì 47121, Italy; Department of Medical and Surgical Sciences (DIMEC), Alma Mater Studiorum, University of Bologna, Via Zamboni, 33, Bologna 40126, Italy; Cardiology Unit, Cardiac Thoracic and Vascular Department, IRCCS Azienda Ospedaliera-Universitaria di Bologna, Bologna, Italy; Department of Medical and Surgical Sciences (DIMEC), Alma Mater Studiorum, University of Bologna, Via Zamboni, 33, Bologna 40126, Italy; Cardiology Unit, Cardiac Thoracic and Vascular Department, IRCCS Azienda Ospedaliera-Universitaria di Bologna, Bologna, Italy; Department of Medical and Surgical Sciences (DIMEC), Alma Mater Studiorum, University of Bologna, Via Zamboni, 33, Bologna 40126, Italy; Cardiology Unit, Cardiac Thoracic and Vascular Department, IRCCS Azienda Ospedaliera-Universitaria di Bologna, Bologna, Italy; Department of Medical and Surgical Sciences (DIMEC), Alma Mater Studiorum, University of Bologna, Via Zamboni, 33, Bologna 40126, Italy; Cardiovascular Division, Morgagni-Pierantoni University Hospital, Via Carlo Forlanini, 34, Forlì 47121, Italy; Department of Medical and Surgical Sciences (DIMEC), Alma Mater Studiorum, University of Bologna, Via Zamboni, 33, Bologna 40126, Italy; Cardiovascular Division, Morgagni-Pierantoni University Hospital, Via Carlo Forlanini, 34, Forlì 47121, Italy; Department of Medical and Surgical Sciences (DIMEC), Alma Mater Studiorum, University of Bologna, Via Zamboni, 33, Bologna 40126, Italy; Cardiology Unit, Cardiac Thoracic and Vascular Department, IRCCS Azienda Ospedaliera-Universitaria di Bologna, Bologna, Italy; Department of Medical and Surgical Sciences (DIMEC), Alma Mater Studiorum, University of Bologna, Via Zamboni, 33, Bologna 40126, Italy; Cardiology Unit, Cardiac Thoracic and Vascular Department, IRCCS Azienda Ospedaliera-Universitaria di Bologna, Bologna, Italy; Department of Medical and Surgical Sciences (DIMEC), Alma Mater Studiorum, University of Bologna, Via Zamboni, 33, Bologna 40126, Italy; Cardiology Unit, Cardiac Thoracic and Vascular Department, IRCCS Azienda Ospedaliera-Universitaria di Bologna, Bologna, Italy; Department of Medical and Surgical Sciences (DIMEC), Alma Mater Studiorum, University of Bologna, Via Zamboni, 33, Bologna 40126, Italy; Cardiovascular Division, Morgagni-Pierantoni University Hospital, Via Carlo Forlanini, 34, Forlì 47121, Italy; Department of Medical and Surgical Sciences (DIMEC), Alma Mater Studiorum, University of Bologna, Via Zamboni, 33, Bologna 40126, Italy; Cardiovascular Division, Morgagni-Pierantoni University Hospital, Via Carlo Forlanini, 34, Forlì 47121, Italy; Department of Medical and Surgical Sciences (DIMEC), Alma Mater Studiorum, University of Bologna, Via Zamboni, 33, Bologna 40126, Italy; Cardiovascular Division, Morgagni-Pierantoni University Hospital, Via Carlo Forlanini, 34, Forlì 47121, Italy; Department of Medical and Surgical Sciences (DIMEC), Alma Mater Studiorum, University of Bologna, Via Zamboni, 33, Bologna 40126, Italy; Cardiovascular Division, Morgagni-Pierantoni University Hospital, Via Carlo Forlanini, 34, Forlì 47121, Italy

**Keywords:** SGLT2 inhibitors, Atrial fibrillation, Heart failure, Heart failure with reduced ejection fraction, Heart failure with preserved ejection fraction, Diastolic dysfunction

## Abstract

**Aims:**

The ability of sodium–glucose co-transporter 2 (SGLT2) inhibitors to prevent atrial fibrillation (AF) has been evaluated in various studies with conflicting results. This study aimed to determine whether SGLT2 inhibitors have a protective effect against AF depending on the baseline clinical condition in which the randomized controlled trials (RCTs) were conducted.

**Methods and results:**

A trial-level meta-analysis was performed including 52 RCTs (112 031 patients) comparing SGLT2 inhibitors with placebo and reporting the number of patients who developed AF in each arm. Risk ratios (RRs) for AF development with 95% confidence intervals (95% CIs) were pooled using a random-effects model. Subgroup analyses were performed by classifying RCTs according to the inclusion criteria of each trial [diabetes, chronic kidney disease, heart failure with reduced ejection fraction (HFrEF), mildly reduced EF (HFmrEF), and preserved EF (HFpEF)]. Overall, SGLT2 inhibitors prevented AF (RR = 0.86, 95% CI 0.77–0.96). In the subgroup analysis, the AF-preventive ability of SGLT2 inhibitors was influenced by HF, being preserved in RCTs recruiting 9141 patients with HFrEF, but not in those recruiting 12 877 subjects with HFmrEF/HFpEF (*P*-value for group difference = 0.01). Meta-regression showed a reduced efficacy of SGLT2 inhibitors in preventing AF when more patients with hypertension or higher EF were enrolled (*P* < 0.01 for both).

**Conclusion:**

Sodium–glucose co-transporter 2 inhibitors prevent AF. Their protective effect was confirmed in the HFrEF subgroup, but not in RCTs recruiting patients with HFmrEF/HFpEF, possibly indicating a different pathophysiology leading to AF among these conditions. However, given the limitations of a trial-level analysis, these findings are exploratory, pending confirmation from patient-level data.

## Introduction

Since the emergence of sodium–glucose co-transporter 2 (SGLT2) inhibitors as drugs capable of reducing hospitalization for heart failure (HF) or cardiovascular death in a wide range of clinical conditions, several studies have sought to demonstrate their benefit on other cardiovascular outcomes.^1-7^ One interesting line of research focuses on evaluating the potential role of SGLT2 inhibitors in the prevention of atrial fibrillation (AF). Initially, this additional benefit of SGLT2 inhibitors appeared to be demonstrated by pooling data from trials testing their efficacy, which were mainly conducted in patients with diabetes mellitus (DM).^8,9^ Nevertheless, more recently, other meta-analyses found no significant AF prevention with SGLT2 inhibitors.^10,11^ However, the inclusion of recent trials conducted only in patients with HF may have jeopardized the results of these latter meta-analyses. Indeed, many factors may contribute to the development of AF, such as hypertension (HTN), DM, obesity, chronic kidney disease (CKD), and HF.^12^ It is important to note that SGLT2 inhibitors may affect these conditions differently with various mechanisms of action, and they may be more effective in preventing AF in certain subgroups of patients.

The aim of this study was to test whether the ability of SGLT2 inhibitors to prevent AF might differ depending on the clinical context and the presence of risk factors for AF.

## Methods

This meta-analysis was registered in the International Prospective Register of Systematic Reviews (PROSPERO; registration number CRD42025639036). The results are reported according to the Preferred Reporting Items for Systematic Reviews and Meta-Analysis guidelines (see Supplementary material online, *Table S1*).

### Search strategy and selection of the studies

PubMed and Cochrane Central Register of Controlled Trials (CENTRAL) were searched for studies for potential inclusion. The complete search strategy is shown in Supplementary material online, *Table S2*, using as main concepts ‘randomised controlled trial’ and ‘SGLT2 inhibitors’. All unique randomized controlled trials (RCTs) comparing SGLT2 inhibitors with placebo and reporting the number of patients who developed AF in both groups published up to 15 January 2025 were included. Exclusion criteria were duplicated populations, non-placebo-controlled trials, and languages other than English. Two reviewers (M.C.A. and A.M.) screened the studies and extracted relevant data. The Cochrane Risk-of-Bias 2 (RoB2) tool was used for quality assessment by two reviewers (N.V. and D.F.). A third reviewer (C.P.) solved disagreements.

### Data extraction

The outcome of interest was the development of AF in the SGLT2 inhibitor and placebo groups. Data were extracted from the main text, supplementary data, published trial protocol, or ClinicalTrials.gov. Studies were classified according to their inclusion criteria. In particular, inclusion criteria relevant for this meta-analysis were DM, CKD, acute or chronic HF, and HF class based on left ventricular ejection fraction (LVEF). The definition of HF was based on the criteria outlined in each study. For the purposes of this study, HF was then classified according to the 2021 European Society of Cardiology guidelines on HF in reduced EF (HFrEF) if LVEF ≤ 40%, mildly reduced EF (HFmrEF) if LVEF is between 41% and 49%, and preserved EF (HFpEF) if LVEF ≥50%.^13^ Studies that recruited patients with HF regardless of LVEF were considered as a separate subgroup. The percentage of patients enrolled in each RCT with DM, CKD, obesity, HTN, coronary artery disease, positive smoking status, HFrEF, HFmrEF or HFpEF, and a history of AF or atrial flutter was also recorded. Finally, mean or median glycated haemoglobin (HbA1c), estimated glomerular filtration rate, body mass index, systolic blood pressure, diastolic blood pressure, and LVEF were collected.

### Statistical analysis

The pooled risk ratio (RR) with a 95% confidence interval (CI) was calculated with a DerSimonian–Laird random-effects model. Heterogeneity was assessed by *I*^2^. Funnel plots and the Egger test were used to assess the risk of small study effects. For subgroup analysis, RCTs were classified as having or not having DM, CKD, and HF as inclusion criteria. In particular, the HF inclusion criterion was further classified according to the timing of HF (acute vs. chronic setting) and LVEF class. In addition, the different SGLT2 inhibitors were considered in the subgroup analysis. Meta-regressions were also performed considering the baseline characteristics of patients included in each RCT (HbA1c, estimated glomerular filtration rate, blood pressure, body mass index, LVEF, and the percentage of patients with DM, CKD, HTN, coronary artery disease, AF, HF, and smoking history). Bubble plots were used to graph the relationship between effect sizes and moderators. Stata SE 18.0 (Stata Corp LLC, College Station, TX, USA) was used for the analyses, and *P*-values <0.05 were considered statistically significant.

## Results

**Figure 1:**
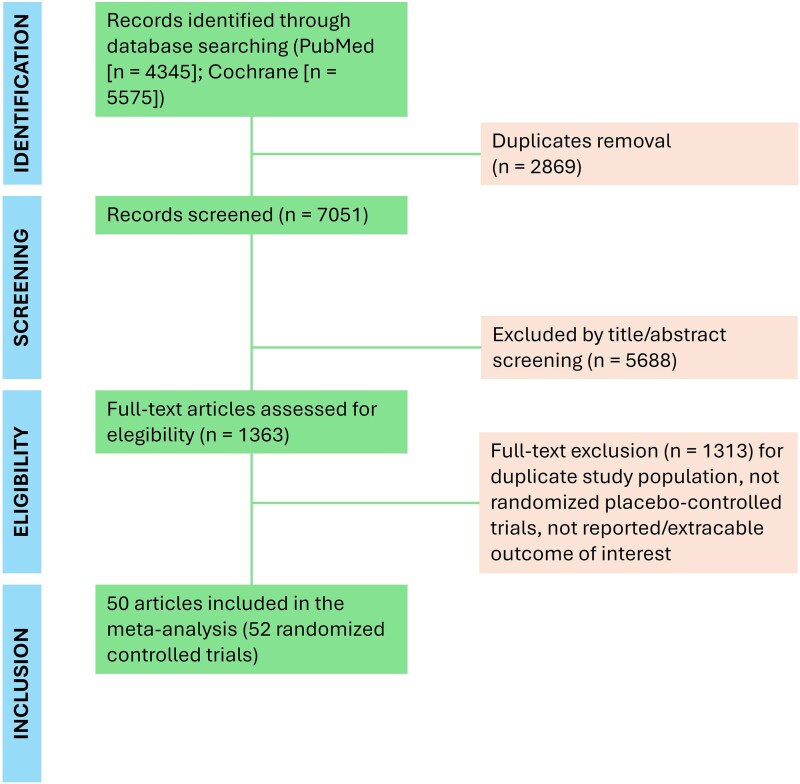
Flowchart of the meta-analysis.

A total of 9920 potential articles published up to 15 January 2025 have been identified. After removing duplicates, screening title and abstract, and excluding non-relevant articles, 52 RCTs were included in the analysis (*Figure 1*), involving 112 031 patients. All RCTs were assessed by the RoB2 tool as having ‘some concerns’ because AF was reported as an adverse event and not systematically assessed, but no relevant concerns were identified (Supplementary material online, *Figure S1*).

The study design and the baseline characteristics of the patients included in the RCTs are shown in *Table 1* and Supplementary material online, *Table S3*. Specifically, 36 RCTs (69.2%) had DM as inclusion criterion, 10 (19.2%) were conducted in patients with CKD, and 13 (25.0%) in patients with HF. Among the included RCTs, 10 had more than one relevant inclusion criterion. Specifically, two studies required both HF and DM, and eight studies included patients with both CKD and DM. In contrast, three RCTs (5.8%) did not require DM, CKD, or HF as inclusion criteria: one was conducted in patients undergoing coronary artery bypass grafting, one in acute myocardial infarction, and one in coronavirus disease 2019. Regarding HF class, five trials included only patients with HFrEF (9141 patients), four RCTs included patients with HFmrEF/HFpEF (12 877 patients), and four trials considered all LVEF classes (2274 patients).

**Table 1:** Summary of the characteristics of included trials

First author or study ID	Year	SGLT2 inhibitor	Sample size	Follow-up duration	Inclusion criteria			
					DM	CKD	HF timing	HF class
Rosenstock	2012	Canagliflozin	386	12 ww	Yes	No	No HF	No HF
Wilding	2012	Dapagliflozin	807	24 ww	Yes	No	No HF	No HF
Bode	2013	Canagliflozin	714	104 ww	Yes	No	No HF	No HF
Inagaki	2013	Canagliflozin	383	14 ww	Yes	No	No HF	No HF
CANTATA-MSU	2013	Canagliflozin	469	52 ww	Yes	No	No HF	No HF
CANTATA-D	2013	Canagliflozin	918	52 ww	Yes	No	No HF	No HF
Bailey	2013	Dapagliflozin	546	102 ww	Yes	No	No HF	No HF
Rosenstock	2013	Empagliflozin	424	12 ww	Yes	No	No HF	No HF
EMPA-REG MONO	2013	Empagliflozin	676	31 ww	Yes	No	No HF	No HF
Yale	2014	Canagliflozin	269	52 ww	Yes	Yes	No HF	No HF
Leiter	2014	Dapagliflozin	965	24 ww	Yes	No	No HF	No HF
EMPA-REG RENAL	2014	Empagliflozin	738	65 ww	Yes	Yes	No HF	No HF
EMPA-REG PIO-TM	2014	Empagliflozin	498	36 ww	Yes	No	No HF	No HF
Bailey	2015	Dapagliflozin	485	102 ww	Yes	No	No HF	No HF
Cefalu	2015	Dapagliflozin	922	24 ww	Yes	No	No HF	No HF
Mathieu	2015	Dapagliflozin	320	52 ww	Yes	No	No HF	No HF
Rosenstock	2015	Empagliflozin	494	82 ww	Yes	No	No HF	No HF
EMPA-REG OUTCOME	2015	Empagliflozin	7020	5 yy	Yes	No	No HF	No HF
CANVAS	2017	Canagliflozin	4327	8 yy	Yes	No	No HF	No HF
CANVAS-R	2017	Canagliflozin	5807	3 yy	Yes	No	No HF	No HF
Softeland	2017	Empagliflozin	332	176 dd	Yes	No	No HF	No HF
inTandem1	2018	Sotagliflozin	793	53 ww	Yes	No	No HF	No HF
VERTIS RENAL	2018	Ertugliflozin	467	54 ww	Yes	Yes	No HF	No HF
VERTIS MET	2018	Ertugliflozin	621	106 ww	Yes	No	No HF	No HF
CREDENCE	2019	Canagliflozin	4397	4.6 yy	Yes	Yes	No HF	No HF
DAPA HF	2019	Dapagliflozin	4736	28.3 mm	No	No	Chronic setting	rEF
DEFINE HF	2019	Dapagliflozin	263	15 ww	No	No	Chronic setting	rEF
DECLARE-TIMI-58	2019	Dapagliflozin	17 143	5.2 yy	Yes	No	No HF	No HF
DELIGHT	2019	Dapagliflozin	293	28 ww	Yes	Yes	No HF	No HF
EMPA-HEARTH	2019	Empagliflozin	97	6 mm	Yes	No	No HF	No HF
DAPA-CKD	2020	Dapagliflozin	4298	39.2 mm	No	Yes	No HF	No HF
EMPA-RESPONSE-AHF	2020	Empagliflozin	79	60 dd	No	No	Acute setting	All LVEF class
EMPEROR-Reduced	2020	Empagliflozin	3726	1047 dd	No	No	Chronic setting	rEF
VERTIS CV	2020	Ertugliflozin	8238	6 yy	Yes	No	No HF	No HF
DARE-19	2021	Dapagliflozin	1229	30 dd	No	No	No HF	No HF
EMPEROR-Preserved	2021	Empagliflozin	5985	1410 dd	No	No	Chronic setting	mrEF or pEF
EMPERIAL-Reduced	2021	Empagliflozin	311	91 dd	No	No	Chronic setting	rEF
EMPERIAL-Preserved	2021	Empagliflozin	315	92 dd	No	No	Chronic setting	mrEF or pEF
SCORED	2021	Sotagliflozin	10 577	30 mm	Yes	Yes	No HF	No HF
SOLOIST-WHF	2021	Sotagliflozin	1216	21.9 mm	Yes	No	Acute setting	All LVEF class
SOTA-CKD4	2021	Sotagliflozin	277	60 ww	Yes	Yes	No HF	No HF
SUGAR-DM-HF	2021	Empagliflozin	105	36 ww	Yes	No	Chronic setting	rEF
CHIEF-HF	2022	Canagliflozin	455	4 mm	No	No	Chronic setting	All LVEF class
DELIVER	2022	Dapagliflozin	6253	42.2 mm	No	No	Chronicsetting^a^	mrEF or pEF
EMPULSE	2022	Empagliflozin	524	127 dd	No	No	Acute setting	All LVEF class
SOCOGAMI	2022	Empagliflozin	42	7 mm	Yes	No	No HF	No HF
SOTA-CKD3	2023	Sotagliflozin	787	60 ww	Yes	Yes	No HF	No HF
EMPA-KIDNEY	2023	Empagliflozin	6609	1171 dd	No	Yes	No HF	No HF
PRESERVED-HF	2023	Dapagliflozin	324	15 ww	No	No	Chronic setting	mrEF or pEF
Halvorsen	2023	Bexagliflozin	317	26 ww	Yes	No	No HF	No HF
DAPA-MI	2024	Dapagliflozin	3972	29 mm	No	No	No HF	No HF
Zarei	2024	Empagliflozin	82	72 hh	No	No	No HF	No HF

CKD, chronic kidney disease; dd, days; DM, diabetes mellitus; hh, hours; HF, heart failure; HFmrEF, heart failure with mildly reduced ejection fraction; HFpEF, heart failure with preserved ejection fraction; HFrEF, heart failure with reduced ejection fraction; ID, identifier; mm, months; SGLT2, sodium–glucose co-transporter 2; ww, weeks.

^a^Included also patients recruited <30 days from decompensation but mainly recruited patients in a chronic setting.

**Figure 2:**
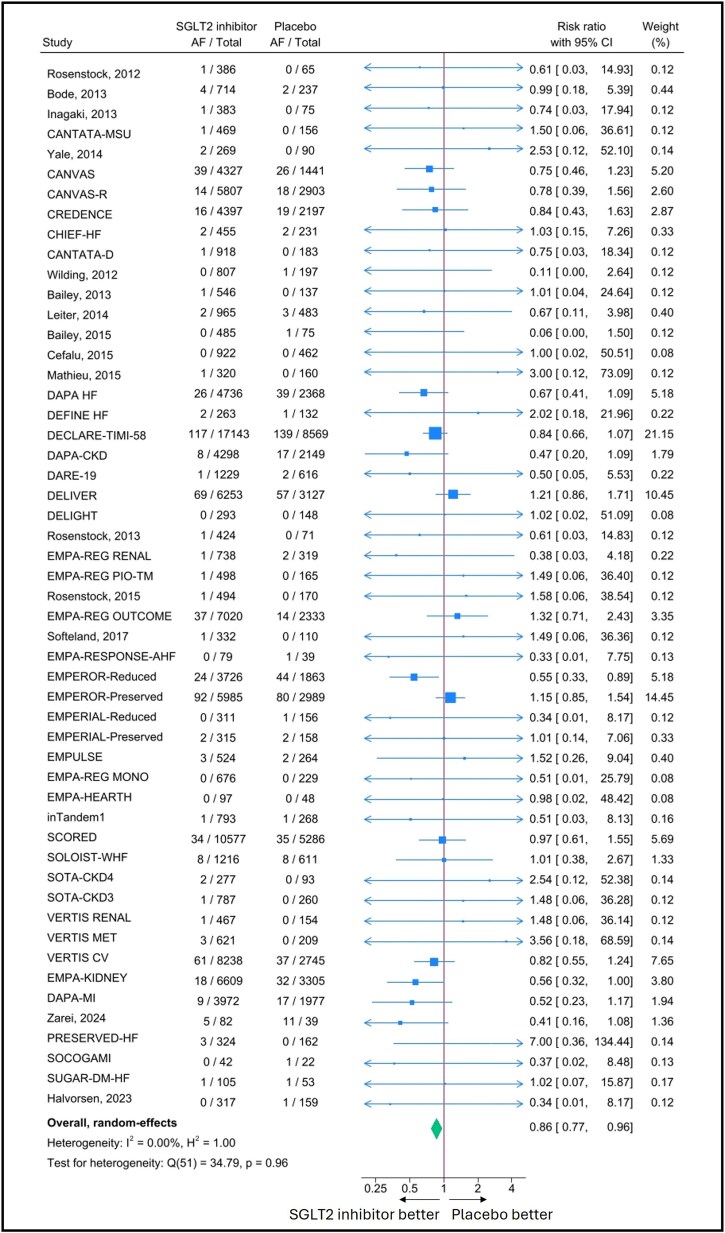
Forest plot illustrating the atrial fibrillation preventive ability of sodium–glucose co-transporter 2 inhibitors. Pooled risk ratios calculated with a DerSimonian–Laird random-effects model. AF, atrial fibrillation; CI, confidence interval; SGLT2, sodium–glucose co-transporter 2.

When considering the 52 RCTs, SGLT2 inhibitors prevented the occurrence of AF (RR = 0.86, 95% CI 0.77–0.96; *I*^2^ = 0%), as shown in *Figure 2*. Funnel plot and Egger test showed no small study effects (Egger test *P* = 0.590; Supplementary material online, *Figure S2*).

**Figure 3:**
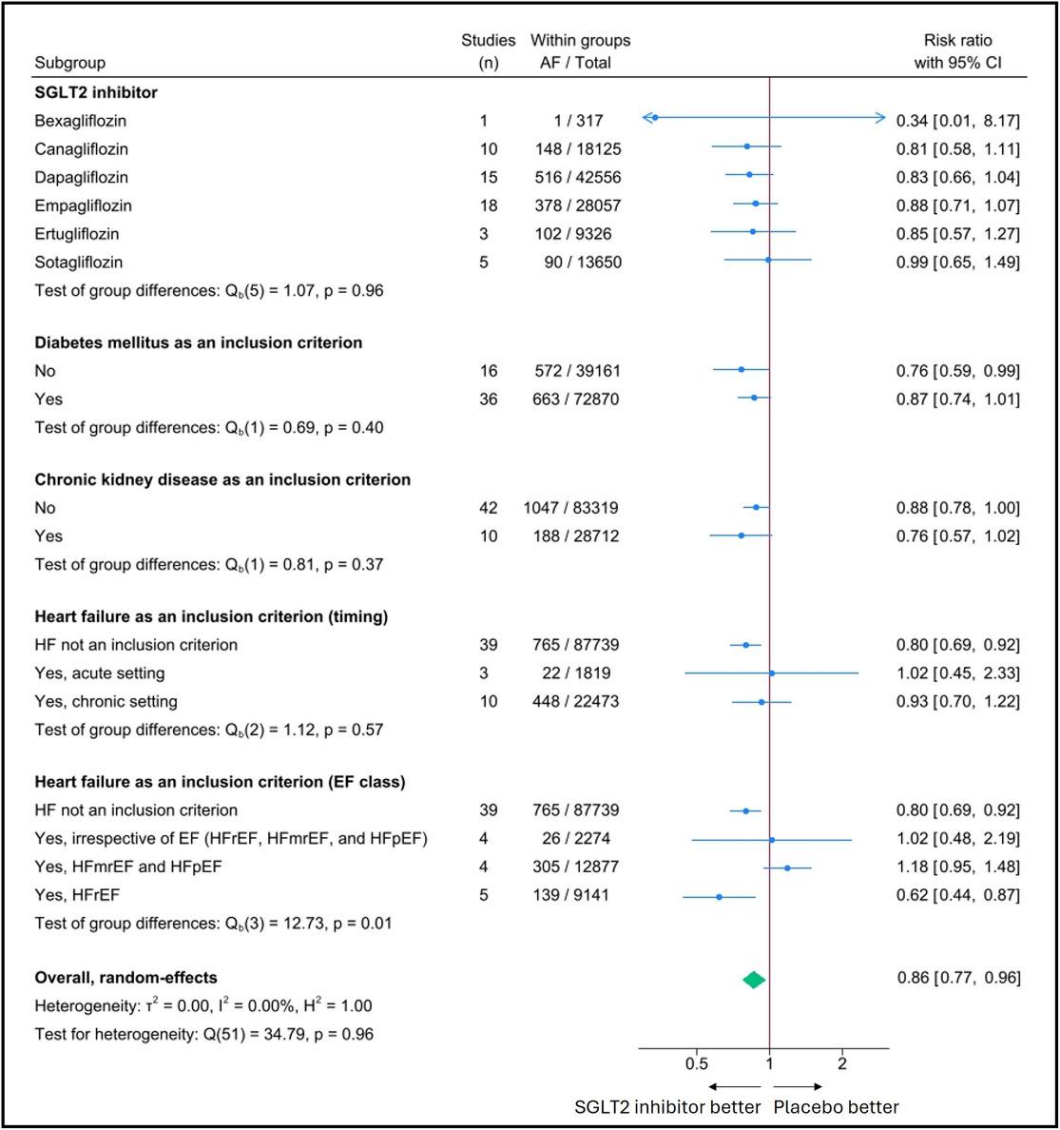
Forest plot illustrating the association between sodium–glucose co-transporter 2 inhibitors and atrial fibrillation occurrences stratified considering the inclusion criteria of the trials. Pooled risk ratios calculated with a DerSimonian–Laird random-effects model. Subgroup analysis showing the protective effect of SGLT2 inhibitors against AF considering the inclusion criteria of the trials, including the presence of diabetes, chronic kidney disease, and heart failure stratified by clinical setting and ejection fraction. AF, atrial fibrillation; CI, confidence interval; EF, ejection fraction; HFmrEF, heart failure with mildly reduced ejection fraction; HFpEF, heart failure with preserved ejection fraction; HFrEF, heart failure with reduced ejection fraction; SGLT2, sodium–glucose co-transporter 2.

There was a significant interaction in the subgroup analysis when RCTs were stratified by HF as an inclusion criterion (*Figure 3*). Specifically, SGLT2 inhibitors were associated with a reduced risk of AF, when HF was not used as an inclusion criterion or when patients with HFrEF were included, but not in patients with HFmrEF or HFpEF (*P*-value for group difference = 0.01). In contrast, no differences were found when RCTs were stratified according to the specific SGLT2 inhibitor or the presence or absence of other specific inclusion criteria, such as DM, CKD, or the timing of HF (acute vs. chronic).

**Figure 4:**
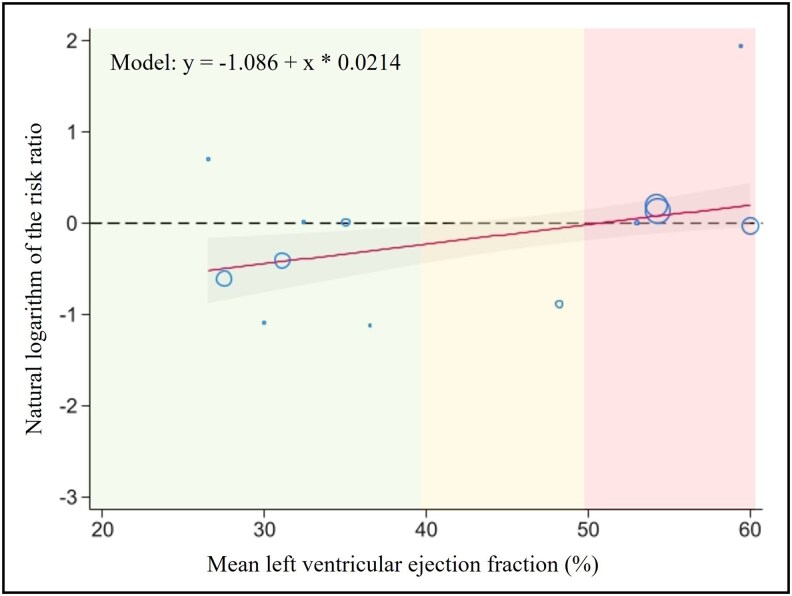
Bubble plot showing the relationship between the risk ratio of developing atrial fibrillation in the sodium–glucose co-transporter 2 inhibitors vs. placebo arms and mean the left ventricular ejection fraction of patients included in each trial. Each trial is represented as a circle, the diameter being representative of its weight. The red line shows the dependence of the natural logarithm of the risk ratio and a mean left ventricular ejection fraction with corresponding 95% confidence intervals (grey area). In this model, a risk ratio = 1 corresponds to mean left ventricular ejection fraction = 50.7%. However, this model is based on trial-level aggregate data and is not intended for individual-level predictions; it only illustrates a trend without implying a hard left ventricular ejection fraction threshold.

Meta-regression showed a reduced efficacy of SGLT2 inhibitors in preventing AF when more patients with HFmrEF or HFpEF were included (*β* = 0.006, *P* = 0.003) and with higher LVEF (*β* = 0.021, *P* = 0.004). The latter corresponds to a 23% loss of AF prevention by SGLT2 inhibitors (i.e. 23% increase in the RR of AF in the SGLT2 inhibitor arm compared with placebo) for every 10% increase in the mean LVEF of the patients included in the RCT. As shown in the bubble plot in *Figure 4*, a neutral effect of SGLT2 inhibitors in preventing AF is estimated when the mean LVEF of the patients included in the RCT approaches 50%. When more patients with known AF or atrial flutter and HTN at baseline were included, the preventive effect of SGLT2 inhibitors on AF was also reduced (*β* = 0.022, *P* = 0.003 and *β* = 0.020, *P* = 0.002, respectively). The other baseline characteristics tested showed no significant effect on outcome (*Table 2*).

**Table 2:** Univariate meta-regression analysis considering the baseline characteristics of patients included in the trials

Baseline characteristic	Studies (*n*)	*β* (95% CI)	*P*-value	Residual *I*^2^ (%)
DM prevalence (%)	52	0.001 (−0.003, 0.005)	0.748	0
Mean HbA1c (mmol/mol)	40	−0.032 (−0.215, 0.151)	0.733	0
CKD prevalence (%)	27	−0.001 (−0.003, 0.005)	0.556	0
Mean eGFR (mL/min)	43	−0.001 (−0.009, 0.007)	0.783	0
Obesity prevalence (%)	6	−0.031 (−0.102, 0.040)	0.390	0
Mean BMI (kg/m^2^)	47	0.057 (−0.019, 0.132)	0.186	0
HTN prevalence (%)	17	0.020 (0.007, 0.032)	0.002	0
Mean SBP (mmHg)	43	0.009 (−0.012, 0.030)	0.397	0
Mean DBP (mmHg)	34	−0.060 (−0.141, 0.020)	0.142	0
Smoking prevalence (%)	10	−0.015 (−0.031, 0.002)	0.077	0
CAD prevalence (%)	13	−0.002 (−0.008, 0.004)	0.519	0
HFrEF prevalence (%)	14	−0.006 (−0.010, −0.002)	0.002	0
HFmr/pEF prevalence (%)	13	0.006 (0.002, 0.010)	0.003	0
Mean LVEF (%)	13	0.021 (0.007, 0.036)	0.004	0
History of AF/AFl prevalence (%)	11	0.022 (0.007, 0.036)	0.003	0

AF, atrial fibrillation; AFl, atrial flutter; BMI, body mass index; CAD, coronary artery disease; CKD, chronic kidney disease; DBP, diastolic blood pressure; DM, diabetes mellitus; eGFR, estimated glomerular filtration rate; HFmrEF, heart failure with mildly reduced ejection fraction; HFpEF, heart failure with preserved ejection fraction; HFrEF, heart failure with reduced ejection fraction; HTN, hypertension; LVEF, left ventricular ejection fraction; SBP, systolic blood pressure.

## Discussion

The main findings of this meta-analysis are the following:

1. The protective role of SGLT2 inhibitors in preventing AF in the overall population included in RCTs was confirmed (RR = 0.86, 95% CI 0.77–0.96).
2. In the subgroup analysis, there was a significant interaction indicating a consistent AF-preventing capacity of SGLT2 inhibitors in the population of patients with HFrEF or in patients enrolled in RCTs not focused on HF, as opposed to RCTs including patients with HFmrEF/HFpEF or HF regardless of LVEF class (*P* for subgroup differences < 0.01).
3. Other possible modulators of the efficacy of SGLT2 inhibitors against AF, beyond higher LVEF, included a history of HTN and previous known AF or atrial flutter.

### The interaction of sodium–glucose co-transporter 2 inhibitors with the heterogeneous pathogenesis of atrial fibrillation

**Figure 5:**
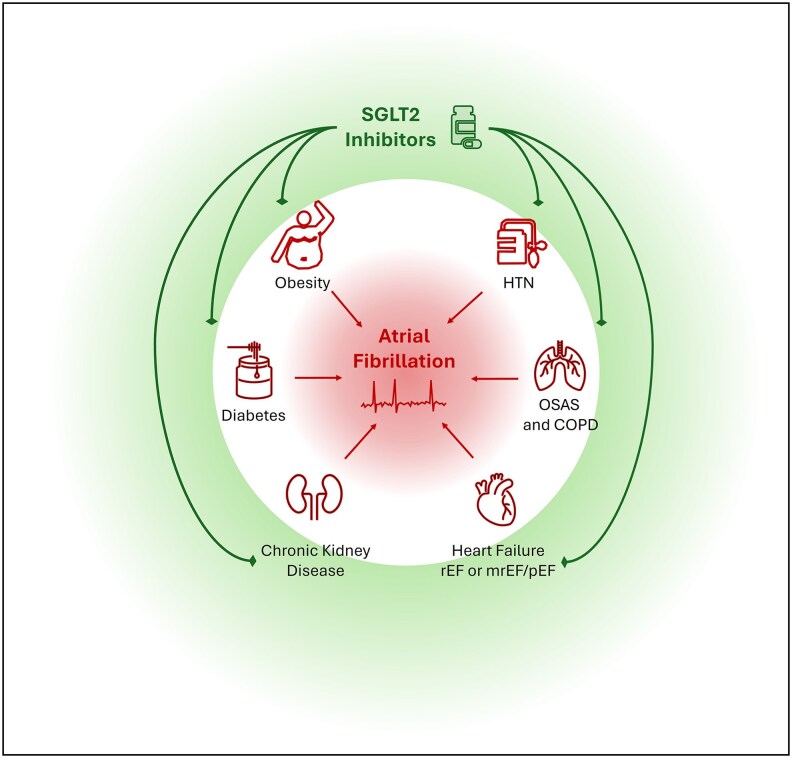
Risk factors for atrial fibrillation and systemic effects of sodium–glucose co-transporter 2 inhibitors. COPD, chronic obstructive pulmonary disease; HTN, hypertension; mrEF, mildly reduced ejection fraction; OSAS, obstructive apnoea syndrome; pEF, preserved ejection fraction; rEF, reduced ejection fraction; SGLT2, sodium–glucose co-transporter 2.

One of the main findings of this meta-analysis is the confirmation of the protective role of SGLT2 inhibitors in the prevention of AF in the overall population included in RCTs. Indeed, from a biological point of view, a Mendelian randomization study demonstrated that SGLT2 inhibition was associated with a reduced risk of both Type 2 DM and AF. Moreover, total concentrations of lipoprotein and HDL particles have been proposed as possible mediators of this effect.^14^ Furthermore, a modulating effect of dapagliflozin on atrial cardiomyocyte excitability has been demonstrated both *in vitro* and in porcine models.^15^ However, AF is a complex condition that can be triggered by many different factors, including atrial wall remodelling, electrolyte imbalance, and fluid overload.^16-18^ It is plausible that SGLT2 inhibitors may prevent AF by acting on only some of these mechanisms (*Figure 5*).^19^ Especially in the heterogeneous population of patients with HF (acute vs. chronic, HFrEF vs. HFpEF), it should be considered that AF develops by different mechanisms and might be differentially susceptible to the preventive effect of SGLT2 inhibitors.^20^

The beneficial effect of SGLT2 inhibitors on cardiovascular death or worsening HF was independent of the presence of AF in the DELIVER (Dapagliflozin Evaluation to Improve the Lives of Patients with Preserved Ejection Fraction Heart Failure) trial.^21^ However, the opposite is not necessarily true, so that SGLT2 inhibitors may not prevent AF regardless of the type of HF. Our results, even if exploratory, show that SGLT2 inhibitors can reduce the development of AF but may not have the same beneficial effect in all types of HF. Similarly, we found that a history of HTN may also impair the AF-preventing ability of SGLT2 inhibitors.

Although a potential role for SGLT2 inhibitors in reducing left ventricular filling pressures, haemodynamic, and atrial dimensions has been demonstrated,^22-25^ much of this evidence is not specific to HFpEF. Diastolic dysfunction is a primary pathological feature in HFpEF and is often associated with other comorbidities such as HTN. Therefore, it is possible that atrial remodelling in HFpEF is too advanced and that AF-generating mechanisms have already developed and are well established. These factors might limit the effectiveness of SGLT2 inhibitors in preventing AF in this population. Conversely, SGLT2 inhibitors may be effective in reducing left atrial filling parameters due to fluid overload. Indeed, a recent subanalysis of the EMPAG-HF (Empagliflozin in Acute Decompensated Heart Failure) study demonstrated that empagliflozin can reduce left atrial volume and left atrial end-systolic volume index after only 5 days of treatment in patients with acute decompensated HF.^26^ In addition, cardiac magnetic resonance-based studies using three-dimensional statistical models of LA shape and feature tracking strain have demonstrated both structural and functional changes that are unique to HFpEF compared with healthy subjects, HFmrEF, and HFrEF.^27,28^ In conclusion, SGLT2 inhibitors may be more beneficial in preventing AF in patients without HF with fewer comorbidities and in those with pure systolic dysfunction (HFrEF) or other conditions where atrial impairment is less pronounced.^29,30^

It is also possible that AF prevention is not a ‘class effect’ but is molecule specific. A nationwide population-based cohort study with propensity score matching by Lim *et al*. found that dapagliflozin had a more pronounced AF-preventive effect than empagliflozin.^31^ Conversely, our subgroup analysis stratifying the RCTs according to the molecule used confirmed the results of other previous studies indicating no interaction between efficacy and the specific SGLT2 inhibitor tested.^8,32^ Furthermore, in the empagliflozin arm of the EMPEROR-Reduced (Empagliflozin Outcome Trial in Patients with Chronic Heart Failure and a Reduced Ejection Fraction) trial the development of AF was significantly lower than in the placebo arm.^33^ However, conclusive evidence is lacking.

### Clinical relevance and future perspectives

It has been argued that even if SGLT2 inhibitors could prevent the occurrence of AF, this could be of little clinical relevance due to the high number needed to treat to prevent a single AF event.^34^ However, although the reduction in the risk of AF with SGLT2 inhibitors appears modest, it should be noted that this benefit may have a secondary impact on AF complications, such as the development of HF, ischaemic stroke, other thromboembolic events, cognitive impairment, and hospitalizations.^12^ Moreover, the management of comorbidities and risk factors is a key step in the new patient-centred AF-CARE approach proposed by European Society of Cardiology guidelines on AF.^12^ SGLT2 inhibitors may well serve as an upstream therapy to indirectly prevent and treat AF, even if they are not properly antiarrhythmic drugs, due to their many mechanisms of action targeting multiple risk factors. Additionally, identifying the specific mechanisms on which SGLT2 inhibitors act could not only improve our understanding of the pathological processes underlying AF, but also facilitate the development of tailored prevention strategies in different clinical contexts.

Catheter ablation of AF represents a potential specific application for SGLT2 inhibitors. An RCT of 80 patients with DM undergoing catheter ablation for paroxysmal AF showed that tofogliflozin had a greater ability to suppress AF recurrence compared with anagliptin.^35^ Lately, Zhao *et al*. confirmed this finding by pooled data from this RCT and three other observational studies.^29^ The DAPA-AF (Use of Dapagliflozin to Reduce Burden of Atrial Fibrillation in Patients Undergoing Catheter Ablation of Symptomatic Atrial Fibrillation; NCT04792190) trial randomized patients undergoing AF catheter ablation to dapagliflozin or placebo and evaluated time spent in AF and AF-related quality of life after the procedure. This trial has been completed and results are not yet published, but it could provide relevant information to expand the indications for SGLT2 inhibitors.

Two RCTs are investigating whether SGLT2 inhibitors can actually prevent AF (NCT04583813, NCT05029115) in diabetic patients with or without HF. In addition, another ongoing trial called DAPA-AF (Dapagliflozin in Patients With Atrial Fibrillation; NCT05174052) will assess the impact of dapagliflozin on AF burden in patients with paroxysmal AF and DM. As suggested by our results, when interpreting the findings of these RCTs, the clinical context in which patients were enrolled should be carefully considered, as it may influence the efficacy of SGLT2 inhibitors.

### Limitations of the study

This is a trial-level meta-analysis, without access to individual patient data and possible lack of relevant information and can therefore only be considered hypothesis generating. In addition, thromboembolic events were not included as an outcome in this meta-analysis; hence, no conclusions can be drawn for this specific endpoint. Another major limitation of our study is represented by the fact that AF was reported as an adverse event in the RCTs and was not systematically searched for, which may have led to an underestimation of the true incidence of AF. The inclusion of patients with HF even in RCTs that did not specifically address this condition cannot be excluded, as only some of the trials considered the presence of HF as an exclusion criterion. However, when reported, the prevalence of HF was used in the meta-regression which confirmed the results of the subgroup analysis. Due to the reduced event rate and sample size in the subgroup analysis, it cannot be excluded that some subgroups were underpowered with a risk of Type II error, so that a significant effect of SGLT2 inhibitors in preventing AF may have been missed in some subgroups.

## Conclusions

SGLT2 inhibitors can prevent AF, particularly in patients without known HF or with HFrEF. However, in our trial-level analysis, this preventive effect was not evident in the subgroup of RCTs recruiting patients with HFmrEF or HFpEF, probably because of too advanced atrial remodelling. Nonetheless, this study is a trial-level meta-analysis and cannot provide definitive evidence on the lack of AF-preventive benefit of SGLT2 inhibitors in patients with HFmEF or HFpEF. It should be considered hypothesis generating until dedicated RCTs or patient-level meta-analyses are conducted to prove causality and elucidate the specific mechanism of action of SGLT2 inhibitors in influencing the different pathways leading to AF in different cardiovascular diseases.

## Supplementary Material

pvaf040_Supplementary_Data

## Data Availability

The data underlying this article will be shared on reasonable request with the corresponding author.
